# Enhanced inflammation in aged mice following infection with *Streptococcus pneumoniae* is associated with decreased IL-10 and augmented chemokine production

**DOI:** 10.1152/ajplung.00141.2014

**Published:** 2015-01-16

**Authors:** Andrew E. Williams, Ricardo J. José, Jeremy S. Brown, Rachel C. Chambers

**Affiliations:** Centre for Inflammation and Tissue Repair, Rayne Institute, University College London (UCL), London, United Kingdom

**Keywords:** pneumonia, neutrophil, chemokine, inflammation, aging

## Abstract

*Streptococcus pneumoniae* is the most common cause of severe pneumonia in the elderly. However, the impact of aging on the innate inflammatory response to pneumococci is poorly defined. We compared the innate immune response in old vs. young adult mice following infection with *S. pneumoniae*. The accumulation of neutrophils recovered from bronchoalveolar lavage fluid and lung homogenates was increased in aged compared with young adult mice, although bacterial outgrowth was similar in both age groups, as were markers of microvascular leak. Aged mice had similar levels of IL-1β, TNF, IFN-γ, IL-17, and granulocyte colony-stimulating factor following *S. pneumoniae* infection, compared with young mice, but increased levels of the chemokines CXCL9, CXCL12, CCL3, CCL4, CCL5, CCL11, and CCL17. Moreover, levels of IL-10 were significantly lower in aged animals. Neutralization of IL-10 in infected young mice was associated with increased neutrophil recruitment but no decrease in bacterial outgrowth. Furthermore, IL-10 neutralization resulted in increased levels of CCL3, CCL5, and CXCL10. We conclude that aging is associated with enhanced inflammatory responses following *S. pneumoniae* infection as a result of a compromised immunomodulatory cytokine response.

the bacterium *Streptococcus pneumoniae* is the leading cause of community-acquired pneumonia (CAP) and the most frequent cause of infectious disease-related admittance to the intensive care unit (32, 33). It has been estimated that *S. pneumoniae* affects 5–6 million people in the USA each year, resulting in 1 million hospitalizations. Furthermore, lower respiratory tract infections account for the highest number of infectious disease-related deaths worldwide, of which *S. pneumoniae* is the predominant etiological agent (4). Age is a major risk factor for *S. pneumoniae* infections, with the majority of cases occurring in the very young or in the elderly (51). Pneumonia often results in the development of septicemia, acute respiratory distress syndrome, and respiratory failure, and these complications are also more common in the elderly (11, 22). However, why the frequency of pneumococcal infection is higher and the disease more severe in the elderly remains poorly defined.

Even though current vaccine regimens have improved clinical outcome in children, age-related immunosenescence hinders vaccine efficacy and enhances disease susceptibility (33, 37). Immunosenescence is a well-recognized phenomenon in the elderly that has been directly related to an increased risk of infectious disease (1, 19). Age-related alterations in the adaptive immune system include an increased production of autoantibodies and decreased production of pneumococcal-specific antibodies (39, 45), decreased T cell proliferative responses (12, 31), and a diminished T cell repertoire (35). These factors are likely to enhance infectious disease susceptibility, exemplified by a higher incidence of infection to a broad spectrum of pathogens, such as influenza virus and *S. pneumoniae* (6, 8, 19), by the reactivation of tuberculosis (23, 38), and a decrease in vaccine efficacy (39).

In contrast to the changes in adaptive immunity, age-related alterations in innate immunity are less well defined. Defects in innate immune cell function maybe associated with age, although innate leukocyte numbers in the elderly reflect those of young adults. Immunosenescence may affect specific functional aspects of innate immune cells (16), such as altered cytokine production and poor responses to inflammatory stimuli (3, 9, 19), including defective TLR2 activation (3). The innate immune system plays a pivotal role in controlling host-pathogen interactions and preventing invasive disease following *S. pneumoniae* colonization of the nasopharynx (27). In particular, alveolar macrophages and recruited neutrophils are thought to be important factors for the control of pneumococcal invasion (26).

The present study aims to address whether the innate immune response of aged mice differs from that of young adult mice in response to *S. pneumoniae* infection. We compared the inflammatory response following infection with *S. pneumoniae* in young adult mice with aged mice and characterized the neutrophil and macrophage response in bronchoalveolar lavage (BAL) fluid and whole lung homogenates. The levels of proinflammatory cytokines, the immunomodulatory cytokine IL-10, and several chemokines were measured. Because of significant differences in IL-10 production between young and aged mice, the effect on pneumococci-induced inflammation, cytokine production, and chemokine levels were assessed following neutralization of IL-10 in young mice. Taken together, these data demonstrate that the innate immune response to *S. pneumoniae* in aged mice is associated with heightened inflammation and diminished IL-10-mediated immunoregulation. These findings have important implications for our understanding of the mechanisms involved in the dysregulation of innate immune responses during aging.

## MATERIALS AND METHODS

### 

#### Bacterial strain and infection model.

The D39 strain (serotype 2) of *S. pneumoniae* was used for all experiments, a virulent strain that is fully infective in mouse lungs. *S. pneumoniae* was cultured on Columbia agar (Oxoid) containing 5% defibrinated horse blood (TSC Biosciences) at 37°C or in Todd-Hewitt medium (Oxoid) containing 5% yeast extract (Oxoid). Growth in liquid medium was assessed by measuring optical density at 580 nm with a spectrophotometer. Bacteria were grown to mid-log phase and numerated by performing serial dilutions and counting colony-forming units (cfu) on Columbia agar.

Young adult female Balb/c mice (6–7 wk old) or aged female Balb/c mice (24 mo old) were housed in identical specified pathogen free conditions, in accordance with the Home Office, UK regulations. Mice were infected intranasally with 50 μl 5 × 10^6^ cfu of *S. pneumoniae*, strain D39 (serotype 2), under general anesthesia (isoflurane). Mice were euthanized 2, 4, 8, 24, or 48 h following infection, depending on the study, and blood, BAL fluid, and whole lungs were removed postmortem.

#### Cell isolation and analysis.

BAL fluid was obtained by making an excision in the trachea and inserting a plastic cannula. The lungs were inflated three times with 750 μl sterile saline and the fluid was withdrawn; 100 μl BAL fluid was used for bacterial colony counts. The BAL fluid was centrifuged at 300 *g* for 5 min to collect the cell pellet. The supernatant was removed and stored at −20°C until analyzed. The cell pellet was resuspended in 500 μl PBS (GIBCO) and total leukocytes were counted by use of a hemocytometer. Cytospin preparations were performed on 200 μl of the cell suspension, centrifuged onto glass slides at 700 rpm for 7 min. Cytospins were then stained with a rapid Romanowski stain (Thermo-Fisher). Differential cell counts were performed by optical microscopy.

Whole lungs were homogenized into a single cell suspension. Lungs were passed through a 40-μm nylon filter (BD Biosciences) with 6 ml of sterile PBS (GIBCO), and 100 μl of the neat cell suspension was used for bacterial colony counts. The cell suspension was centrifuged at 300 *g* for 5 min to obtain a cell pellet. The lung supernatant was removed and stored at −20°C until used. The cell pellet was resuspended in 1 ml of sterile PBS and counted by use of a hemocytometer.

#### Bacterial culture.

Bacteria recovered from BAL fluid, whole lung homogenates, and blood were counted by performing serial dilutions and were cultured on Columbia agar containing 5% defibrinated horse serum as described. Cultures were incubated at 37°C for 18 h; cfu were counted and numbers transformed into log-10 values.

#### Flow cytometric analysis of lung cells.

Leukocyte recruitment into lung tissue was analyzed by flow cytometry, and 100 μl containing 2 × 10^5^ cells from homogenized lung tissue were used for each stain. Cells were centrifuged at 400 *g* for 2 min and resuspended in staining medium containing PBS, 4% bovine serum albumin (Sigma), and 0.1% sodium azide (Sigma). The following antibodies specific to mouse surface markers were used: Ly6G-PE (clone 1A8, BD Biosciences), CD11b-APC (BD Biosciences), and CD11c-PerCP (BD Biosciences). Isotype controls (BD Biosciences) and Fluorescent Minus One (FMO) control stains were performed. Flow cytometry analysis was performed on a FACSVerse (BD Biosciences) and analyzed with FlowJo software (Tree Star). The total leukocyte population was gated based on FSc and SSc and neutrophils were analyzed based on their specific expression of Ly6G and CD11b (Ly6G^high^CD11b^hi^CD11c^lo^ population). Differential cell counts were calculated based on the percentage of neutrophils within the total leukocyte population.

#### Protein, cytokine, and chemokine analysis.

BAL fluid was used to analyze endothelial-epithelial barrier permeability and surrogate markers of coagulopathy. Endothelial-epithelial barrier permeability was measured by analyzing the amount of serum albumin recovered in BAL fluid with an ELISA (Bethyl Laboratories). Coagulopathy was assessed by measuring thrombin-anti-thrombin (TAT) complexes (EnymeResearch) or activated protein C (APC) (Antibodies Online) recovered from BAL fluid by ELISA. Neutrophil elastase (ELA2) was measured by ELISA (Caltag-MedSystems) and the ELA2-to-neutrophil ratio for BAL fluid was calculated. The levels of cytokines and chemokines were analyzed in supernatant derived from whole lung homogenates. The proinflammatory cytokines IL-1β, TNF, IFN-γ, IL-17, and granulocyte colony-stimulating factor (G-CSF) and the immunomodulatory cytokine IL-10 were all measured by ELISA (PeproTech). Chemokines were measured by use of a multiarray ELISA platform (Qiagen).

#### IL-10 neutralization in vivo.

Mice were intranasally infected with 50 μl 5 × 10^6^ cfu of *S. pneumoniae* strain D39 (serotype 2). In addition, the inoculum contained 10 μg of functional grade anti-IL-10 antibody per mouse (eBioscience) or poly-Ig control (PeproTech). This antibody has been shown to inhibit 50% the biological activity of IL-10 (at a concentration of 4 ng/ml of antibody in the presence of 1 ng/ml antigen), as reported by the manufacturers. Animals were euthanized 2, 4, 8, 24, or 48 h following challenge, as described, and BAL fluid, lung tissue, and blood were removed postmortem.

#### Statistical analysis.

Data were analyzed by one-way analysis of variance (ANOVA) with Newman-Keuls posttest to compare all groups, or with two-way ANOVA across time courses, or with a Student's *t*-test when comparing two groups. A *P* value of less than 0.05 was considered significant.

## RESULTS

### 

#### Enhanced inflammation in aged mice following S. pneumoniae infection.

**Fig. 1.**
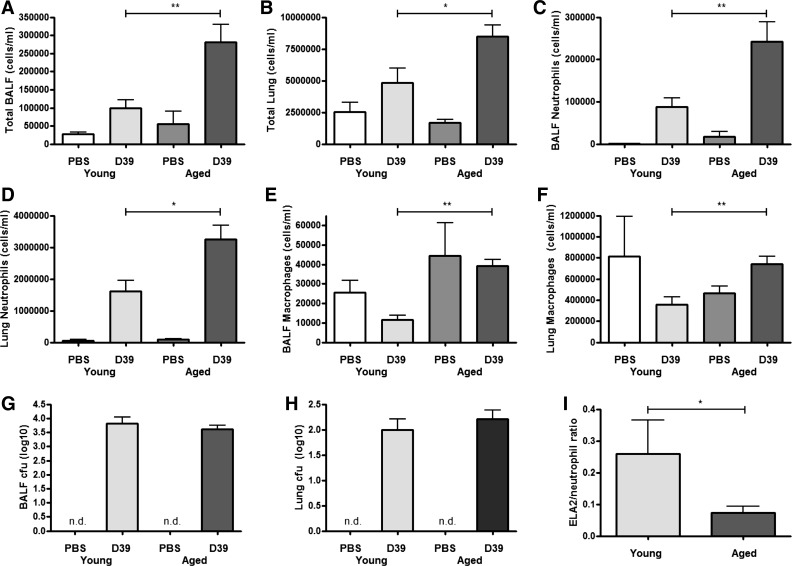
Differential inflammatory response between young and aged mice. Young adult Balb/c mice (6–7 wk of age, *n* = 4–6 per group) or aged Balb/c mice (24 mo of age, *n* = 3–5 per group) were intranasally challenged with 5 × 10^6^ cfu *Streptococcus pneumoniae* strain D39 (serotype 2) and euthanized 24 h later. Total cells were recovered from bronchoalveolar lavage fluid (BALF; *A*) and lung tissue (*B*). Differential cell counts were performed on BALF cytospin preparations or flow cytometry analysis was performed on whole lung cell homogenates. Analysis determined total neutrophil (*C* and *D*) and macrophage numbers (*E* and *F*) in BALF and lung tissue, respectively. Bacteria recovered from BALF (*G*) and whole lung homogenates (*H*) were cultured on Columbia agar containing 5% defibrinated horse serum. Cultures were incubated at 37°C for 18 h. Colony-forming units (cfu) were counted and numbers were transformed into log10 values. Neutrophil elsatse-2 (ELA2) was measured in the BALF of infected mice and the ELA2-to-neutrophil number ratio calculated (*I*). Statistical analysis was performed by using an ANOVA with Newman-Keuls posttest to compare all groups (**P < 0.001; *P < 0.05).

To assess whether there are age-related differences in the innate immune response to *S. pneumoniae*, young adult mice (6–7 wk) or aged mice (24 mo) mice were challenged with 5 × 10^6^ cfu of the D39 *S. pneumoniae* strain and euthanized 24 h later. Infection with *S. pneumoniae* resulted in an acute inflammatory response in both young and aged animals. The total number of cells recovered from the BAL fluid (Fig. 1*A*) and from whole lung homogenates (Fig. 1*B*) of aged mice were higher than those recovered from young mice. Differential cell counts revealed that the number of neutrophils isolated from the BAL fluid and lung homogenates were significantly higher in aged mice compared with young mice following infection (Fig. 1, *C* and *D*), indicating that aged mice mount a heightened innate inflammatory response to *S. pneumoniae*. Furthermore, although macrophage numbers were slightly increased in aged mice following infection, macrophage numbers at baseline were similar between young and aged mice in both BAL fluid and lung homogenates (Fig. 1, *E* and *F*).

We next determined the effect of age on the ability of the host to control bacterial outgrowth. The bacterial cfu recovered from the BAL fluid of aged and young mice were comparable (Fig. 1*G*), as was the bacterial burden based on cfu recovered from whole lung homogenates (Fig. 1*H*). In addition, neither young mice nor aged mice developed septicemia after 24 h, since no bacteria were recovered from blood (data not shown).

Considering that aged mice had significantly increased neutrophil numbers in the BAL fluid compared with young mice, but no corresponding decrease in bacterial cfu, we next assessed neutrophil function by measuring neutrophil elastase production in BAL fluid of infected mice. Aged mice had a decrease in the ELA2-to-neutrophil ratio compared with young mice (Fig. 1*I*), indicating reduced capacity to produce ELA2 in aged neutrophils.

#### Aged mice do not exhibit increased barrier permeability.

**Fig. 2.**
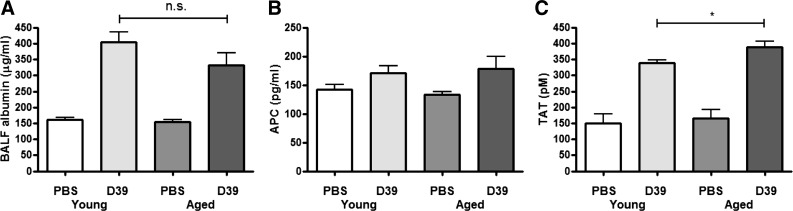
Analysis of endothelial-epithelial barrier integrity. Young adult Balb/c mice (6–7 wk of age, *n* = 4–6 per group) or aged Balb/c mice (24 mo of age, *n* = 3–5 per group) were intranasally challenged with 5 × 10^6^ cfu *S. pneumoniae* strain D39 (serotype 2) and euthanized 24 h later. BALF was recovered and serum albumin (*A*), activated protein C (APC; *B*), and thrombin-anti-thrombin complexes (TAT; *C*) measured by ELISA. Statistical analysis was performed by using an ANOVA with Newman-Keuls posttest to compare all groups (*P < 0.05); n.s., not significant.

We next aimed to investigate whether aged mice display evidence of increased endothelial-epithelial barrier permeability or coagulopathy. Serum albumin levels, recovered from BAL fluid, increased following *S. pneumoniae* infection in both young and aged mice, although there was no difference between the two age groups (Fig. 2*A*), indicating that increased neutrophil influx does not result in increased barrier permeability. To assess potential differences in coagulation, APC and TAT were measured in BAL fluid. There was no difference in APC levels in young compared with old mice (Fig. 2*B*). However, aged mice had a small but significant increase in BAL fluid TAT levels (Fig. 2*C*), indicating that aged animals exhibit an enhanced procoagulant state.

#### Decreased IL-10 production in aged mice.

**Fig. 3.**
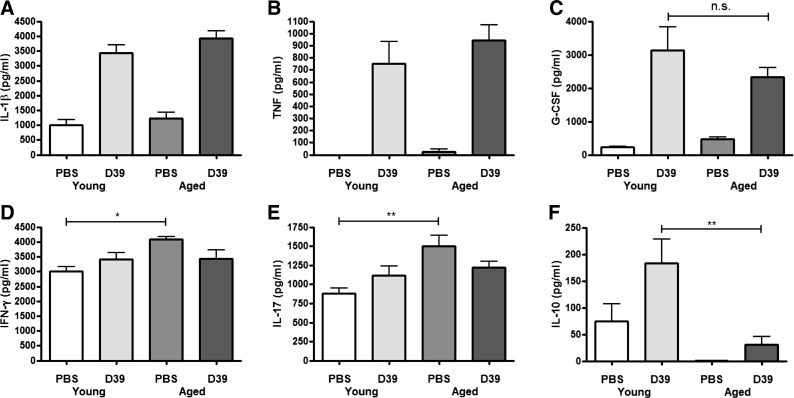
Aged mice produce less IL-10 following *S. pneumoniae* infection. Young adult Balb/c mice (6–7 wk of age, *n* = 4–6 per group) or aged Balb/c mice (24 mo of age, *n* = 3–5 per group) were intranasally challenged with 5 × 10^6^ cfu *S. pneumoniae* strain D39 (serotype 2) and euthanized 24 h later. Lung tissue was removed postmortem and homogenized, the cell pellet was centrifuged, and the supernatant was removed. The levels of IL-1β (*A*), TNF (*B*), granulocyte colony-stimulating factor (G-CSF; *C*), IFN-γ (*D*), IL-17 (*E*), and IL-10 (*F*) in lung homogenates were all measured by ELISA. Statistical analysis was performed by using an ANOVA with Newman-Keuls posttest to compare all groups (*P < 0.05).

To determine whether the heightened inflammatory response in aged mice was associated with an increase in pulmonary cytokine levels, we measured several proinflammatory cytokines and the immunomodulatory cytokine IL-10 in lung tissue. Although the levels of IL-1β, TNF, and G-CSF increased following infection with *S. pneumoniae* in both young and aged mice, no differences between the two age groups were detected (Fig. 3, *A*–*C*). The levels of IFN-γ and IL-17 did not significantly increase following infection, although baseline levels were higher in aged mice compared with young (Fig. 3, *D* and *E*). In contrast, baseline levels of IL-10 were higher in young compared with aged mice (Fig. 3*F*), whereas young mice exhibited increased IL-10 levels following infection with *S. pneumoniae*. Taken together, these data suggest that the heightened inflammatory response in aged mice is associated with reduced IL-10 levels rather than an increased proinflammatory cytokine response.

#### Elevated chemokine production in aged mice.

**Fig. 4.**
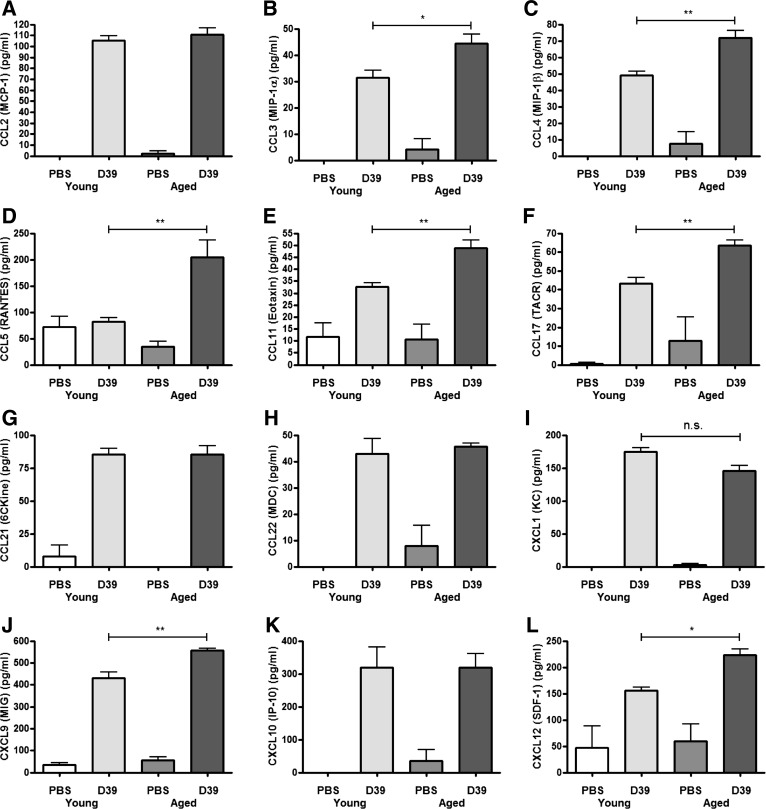
Aged mice have a dysregulated chemokine response. Young adult Balb/c mice (6–7 wk of age, *n* = 4–6 per group) or aged Balb/c mice (24 mo of age, *n* = 3–5 per group) were intranasally challenged with 5 × 10^6^ cfu *S. pneumoniae* strain D39 (serotype 2) and euthanized 24 h later. Lung tissue was removed postmortem and homogenized, the cell pellet was centrifuged, and the supernatant was removed. The levels of CCL2 (*A*), CCL3 (*B*), CCL4 (*C*), CCL5 (*D*), CCL11 (*E*), CCL17 (*F*), CCL21 (*G*), CCL22 (*H*), CXCL1 (*I*), CXCL9 (*J*), CXCL10 (*K*), and CXCL12 (*L*) were measured by multianalyte ELISA array. Statistical analysis was performed by using an ANOVA with Newman-Keuls posttest to compare all groups (**P < 0.001; *P < 0.05).

We next measured the levels of several chemokines in lung tissue. Aged animals exhibited enhanced production of a number of chemokines following infection with *S. pneumoniae* compared with young animals (Fig. 4). Baseline levels of these chemokines were low or undetectable in PBS-instilled mice regardless of age. Increased production of CCL3 (MIP-1a), CCL4 (MIP-1b), CCL5 (RANTES), CCL11 (Eotaxin), CCL17 (TARC), CXCL9 (MIG), and CXCL12 (SDF-1) were detected in aged mice compared with young mice (Fig. 4), suggesting that the increased neutrophil margination into the lungs of aged mice may be associated with increased levels of several chemokines. Of note, although the known neutrophil chemoattractants CXCL1 (KC) and CCL2 (MCP-1) increased following infection, the levels were similar in aged vs. young mice (Fig. 4, *A* and *I*).

#### IL-10 modulates the inflammatory response to S. pneumoniae.

**Fig. 5.**
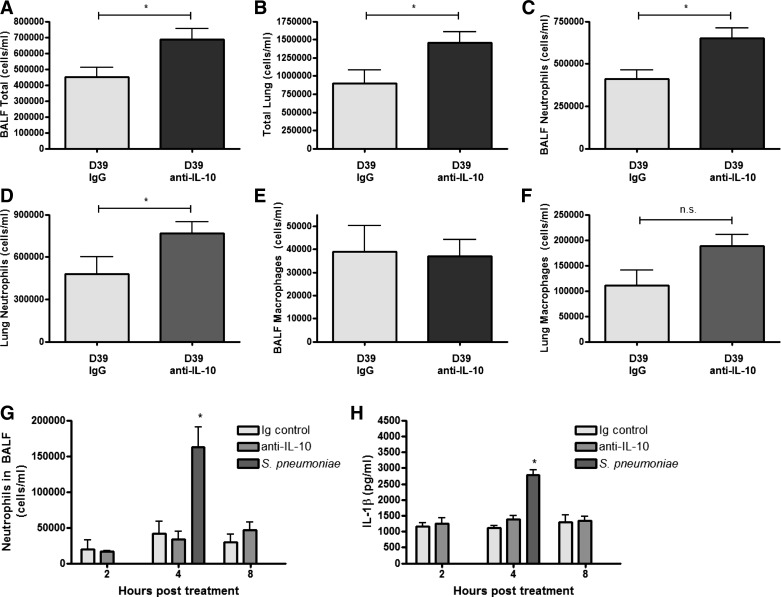
IL-10 modulates neutrophilic inflammation following *S. pneumoniae* infection. Young adult Balb/c mice (6–7 wk of age, *n* = 5 per group) were intranasally challenged with 5 × 10^6^ cfu *S. pneumoniae* strain D39 (serotype 2), treated with nonspecific poly-IgG control antibody or anti-IL-10 neutralizing antibody, and euthanized 24 h later. Total cells were recovered from BALF (*A*) and lung tissue (*B*). Differential cell counts were performed on BALF cytospin preparations or flow cytometry analysis was performed on whole lung cell homogenates. Analysis determined total neutrophil (*C* and *D*) and macrophage numbers (*E* and *F*) in BALF and lung tissue, respectively. Neutrophil numbers (*G*) and IL-1β levels (*H*) were determined at 2, 4, and 8 h after anti-IL-10 or poly-IgG control antibody treatment in the absence of infection. Statistical analysis was performed by using a Student's *t*-test when comparing 2 groups or 2-way ANOVA to compare all groups (*P < 0.05).

We next aimed to determine the relationship between IL-10 and the modulation of the inflammatory response following *S. pneumoniae* infection. Young adult mice were infected with 5 × 10^6^ cfu of *S. pneumoniae* with or without 10 μg of a neutralizing anti-IL-10 antibody given within the intranasal challenge volume. Compared with IgG control antibody-treated mice, those that received anti-IL-10 had an increase in total cell (Fig. 5, *A* and *B*) and neutrophil numbers (Fig. 5, *C* and *D*) recovered from the BAL fluid and lung tissue, although macrophage numbers were similar (Fig. 5, *E* and *F*). These data indicate that IL-10 modulates the magnitude of the neutrophil influx into the lungs of *S. pneumoniae*-challenged mice.

To assess whether the anti-IL-10 neutralizing antibody was inducing inflammation itself, mice were treated with anti-IL-10 antibody or control antibody via the intranasal route in the absence of concurrent bacterial infection. No differences in the total cell or neutrophil numbers (Fig. 5*G*) were observed 2, 4, or 8 h after treatment. Furthermore, no differences in IL-1β production between control antibody and anti-IL-10 antibody were detected (Fig. 5*H*) indicating an absence of inflammation.

#### No decrease in bacterial outgrowth in the absence of IL-10.

**Fig. 6.**
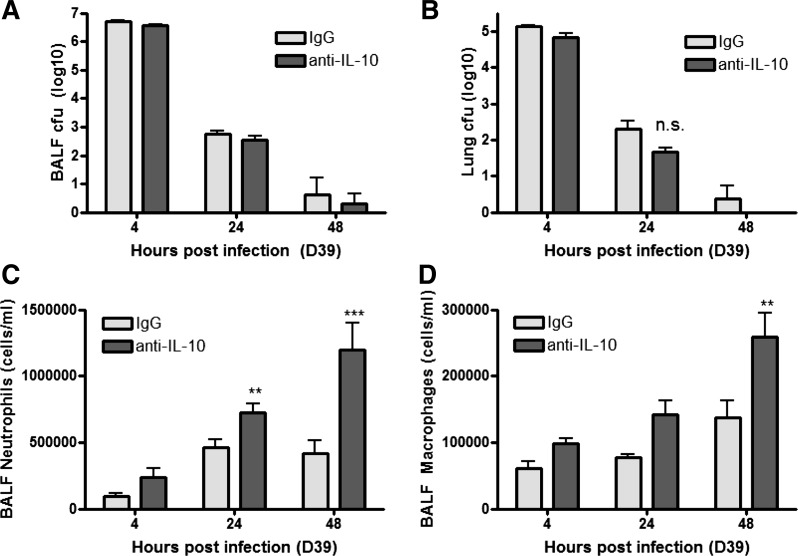
IL-10 has no effect on bacterial clearance. Young adult Balb/c mice (6–7 wk of age, *n* = 5 per group) were intranasally challenged with 5 × 10^6^ cfu *S. pneumoniae* strain D39 (serotype 2), treated with nonspecific poly-IgG control antibody or anti-IL-10 neutralizing antibody, and euthanized 4, 24 or 48 h later. Bacteria recovered from BALF (*A*) and whole lung homogenates (*B*) were cultured on Columbia agar containing 5% defibrinated horse serum. Cultures were incubated at 37°C for 18 h. The cfu were counted and numbers were transformed into log10 values. Differential cell counts were performed on BALF cytospin preparations and neutrophil (*C*) and macrophage (*D*) total cell counts calculated. Statistical analysis was performed by using a 2-way ANOVA comparing all groups (***P < 0.001; **P < 0.005).

We next measured *S. pneumoniae* recovered from BAL fluid and lung homogenates following treatment with anti-IL-10 antibody. No difference in bacterial counts was observed in recovered BAL fluid (Fig. 6*A*) or lungs (Fig. 6*B*) at 4, 24, or 48 h compared with control mice following infection with *S. pneumoniae*, suggesting that the control of bacterial outgrowth is independent of IL-10. Bacteremia was not detected at any of the time points (data not shown), measured by cfu counts from the blood. Furthermore, neutralization of IL-10 at the point of infection with *S. pneumoniae* resulted in elevated neutrophil and macrophage numbers in the BALF up to 48 h postinfection compared with control antibody-treated mice (Fig. 6, *C* and *D*), despite a significant decrease in bacterial numbers within the BAL fluid and lung at this time point.

**Fig. 7.**
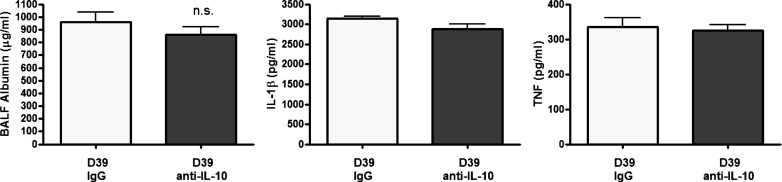
IL-10 does not affect leak, TNF, or IL-1β levels. Young adult Balb/c mice (6–7 wk of age, *n* = 5 per group) were intranasally challenged with 5 × 10^6^ cfu *S. pneumoniae* strain D39 (serotype 2), treated with nonspecific poly-IgG control antibody or anti-IL-10 neutralizing antibody, and euthanized 24 h later. BALF was recovered and serum albumin (*left*) was measured by ELISA. Lung tissue was removed postmortem and homogenized, the cell pellet was centrifuged, and the supernatant was removed. The levels of IL-1β (*middle*) and TNF (*right*) in lung homogenates were measured by ELISA. Statistical analysis was performed by using a Student's *t*-test (*P < 0.05).

In addition, IL-10 treatment did not alter barrier permeability as determined by serum albumin levels in recovered BAL fluid (Fig. 7*A*) 24 h after infection with *S. pneumoniae*. No differences in the levels of IL-1β or TNF were detected following anti-IL-10 treatment compared with IgG controls (Fig. 7, *B* and *C*), suggesting that IL-10 is not involved in modulating these cytokines, consistent with the data obtained for young vs. aged mice.

#### IL-10 regulates CCL3, CCL5, and CXCL10 production.

**Fig. 8.**
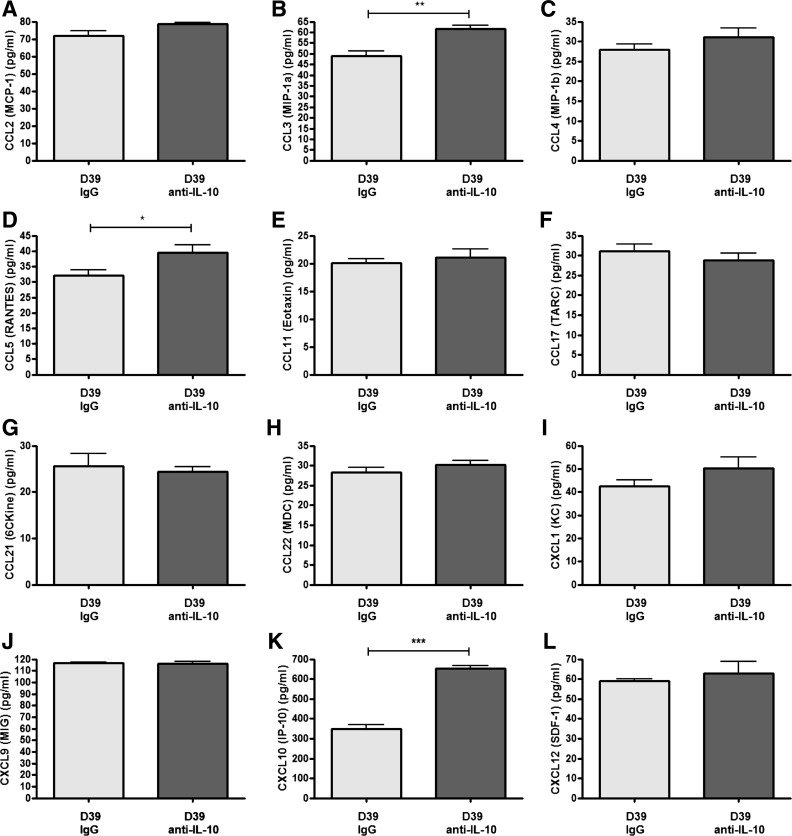
IL-10 modulates the release of CCL3, CCL5, and CXCL10. Young adult Balb/c mice (6–7 wk of age, *n* = 5 per group) were intranasally challenged with 5 × 10^6^ cfu *S. pneumoniae* strain D39 (serotype 2), treated with nonspecific poly-IgG control antibody or anti-IL-10 neutralizing antibody, and euthanized 24 h later. Lung tissue was removed postmortem and homogenized, the cell pellet was centrifuged, and the supernatant was removed. The levels of CCL2 (*A*), CCL3 (*B*), CCL4 (*C*), CCL5 (*D*), CCL11 (*E*), CCL17 (*F*), CCL21 (*G*), CCL22 (*H*), CXCL1 (*I*), CXCL9 (*J*), CXCL10 (*K*), and CXCL12 (*L*) were measured by multianalyte ELISA array. Statistical analysis was performed by using a Student's *t*-test (***P < 0.0001; **P < 0.001; *P < 0.05).

To determine whether IL-10 has a modulatory effect on chemokine production, the levels of several chemokines were assessed following infection. Of all the chemokines tested, treatment with anti-IL-10 neutralizing antibody only led to a significant increase in the pulmonary levels of CCL3, CCL5, and CXCL10 (Fig. 8). Increased expression of CCL3 and CCL5 following anti-IL-10 treatment is consistent with the enhanced chemokine response in aged mice compared with young mice (Fig. 4). These data indicate that IL-10 modulates the expression of a subset of chemokines associated with the innate immune response following infection with *S. pneumoniae*.

## DISCUSSION

*S. pneumoniae* is the leading cause of CAP, accounting for more than 1 million hospitalizations in the US and 1 million childhood deaths worldwide each year (2). Although vaccination has been successful at reducing disease burden in infants, immunocompromised patients are still at risk of developing invasive disease (20, 40). In particular, the elderly population (>65 years of age) represents a substantial and growing group of individuals who are at particular risk of developing life-threatening pneumococcal pneumonia (6, 29). As global demographics shift toward an expanding elderly population, understanding host-pathogen interactions in aging individuals is therefore paramount.

In the present study we specifically focused on the acute phase of *S. pneumoniae* infection and compared the innate immune response in aged vs. young mice. Aged mice exhibited an enhanced inflammatory response to the pneumococcus, as demonstrated by higher numbers of total leukocytes, neutrophils, and macrophages recovered from the BAL fluid and lung tissue following challenge. The enhanced neutrophil response may be of particular relevance to the pathogenesis of disease due to the pivotal role that these cells play in host defense (30). The increased macrophage response in aged mice further suggests a dysregulated innate immune response in these animals. Moreover, alveolar macrophages often marginate and adhere to the epithelium during acute inflammatory reactions (25), as seen in young mice in this study, a process that may be defective in aged mice. Indeed, macrophages from aged animals have previously been shown to be less responsive to inflammatory signals (24). Alternatively, the increased number of macrophages in aged mice may be due to the underlying proinflammatory state, which may result in increases in both resident and recruited macrophage populations. However, it is accepted that there are some limitations in using aged mice to model responses in elderly humans, as not all age-related changes can be modeled accurately. For example, aged mice are not exposed to a lifetime of environmental insults and do not possess many of the comorbidities associated with aged humans. Despite these caveats, this model does represent a useful means of studying acute inflammation in the lung to understand how the aging process affects innate immune responses to bacterial pathogens.

Taking into account the enhanced recruitment of neutrophils in aged mice, we next assessed how this would affect bacterial clearance. Despite higher neutrophil numbers, pneumococcal clearance from the lungs was not enhanced in aged compared with young mice. This may be explained by the finding that the relationship between neutrophil recruitment and pathogen clearance is not linear. An increase in neutrophil numbers therefore does not necessarily mean that the pneumococcus will be cleared more effectively. This may also reflect discrepancies between neutrophil accumulation and normal function, whereby a decline in bacterial killing may further compromise host defense (18). Neutrophil function has previously been demonstrated to be compromised in aged mice, so that bacteria are cleared less efficiently. Indeed, neutrophils from aged mice have a reduced phagocytic killing potential (46) and are less capable of generating reactive oxygen species (53). Neutrophils from aged humans also have reduced phagocytic activity (13), which may be related to lower CD16 expression (5) or to defective Fcγ-receptor-mediated uptake of opsonized microbes (15). In addition, elderly neutrophils are less effective at phagocytosing and killing pneumococci opsonized by immunoglobulin (46). In the present study we further demonstrate that ELA2 production per neutrophil is compromised in aged mice, which could explain the unaffected bacterial counts, despite increased cell numbers, in our model. It may be the case that aged individuals recruit more neutrophils into sites of infection to compensate for a reduced antimicrobial capacity.

Because of the potential for enhanced tissue damage as a result of excessive neutrophilia, we next assessed endothelial-epithelial barrier permeability in young and aged mice. Although aged mice recruited more neutrophils, endothelial-epithelial barrier permeability, as measured by serum albumin levels in the BAL fluid, was not correspondingly increased. There is some debate as to whether neutrophil migration directly contributes to barrier permeability, whereas it has been suggested that the activation of neutrophils is required for bystander tissue damage to occur (10, 47, 54). Although barrier integrity was compromised following *S. pneumoniae* challenge, this was not directly associated with the observed neutrophil influx. This apparent paradoxical observation may be accounted for by the poor functionality of neutrophils derived from aged compared with young animals (46, 53). Indeed, the release of proteinases, such as matrix metalloproteinases, ELA2, and other granule-derived enzymes, has been shown to contribute, and even be required for, the tissue damage associated with neutrophil recruitment into the lung (55–57). We further found that BAL fluid TAT complex levels were elevated in aged compared with young mice, suggesting that aging is associated with an enhanced pulmonary procoagulant state in these animals. Coagulation markers in the circulation have been shown to increase in patients hospitalized with CAP, although no correlations were made with clinical outcome (28, 34).

To account for the heightened recruitment of leukocytes into the lungs following *S. pneumoniae* challenge in aged mice, we next analyzed the levels of several proinflammatory cytokines. We found that IL-1β, TNF, G-CSF, IFN-γ, and IL-17 levels were similar in young and old mice. However, IL-10 levels were significantly lower in aged mice compared with young mice at baseline levels and in response to *S. pneumoniae* infection. IL-10 is an immunosuppressive cytokine capable of modulating both innate and adaptive immune responses. Initially recognized as a regulatory cytokine produced by T cells, it is now known to be expressed by a variety of cells, including macrophages, neutrophils, and epithelial cells and has diverse modulatory effects on the immunopathology induced by infectious microorganisms (42). However, aging is associated with chronic inflammation, termed inflammaging (14), which may modulate the phenotype of both immune and nonimmune cells. However, the cell-specific changes in propensity for IL-10 production remain poorly defined and findings have often been paradoxical. For example, the T cell system in aged humans is biased toward an inflammatory Th17 phenotype under basal condition but shifts toward a regulatory T cell phenotype upon stimulation, indicating that an imbalance exists between pro- and anti-inflammatory immune responses (43). There may also be tissue-specific differences in the aging immune system, as exemplified by decreased production of TGF-β and IL-10 in the gut mucosa (41). The reduced levels of IL-10 in aged mice may therefore suggest a lack of immunoregulation in the lungs following bacterial infection, thereby contributing to enhanced recruitment of neutrophils and inflammatory disease.

To further evaluate the contribution of IL-10 on the acute phase of *S. pneumoniae* infection, we inhibited IL-10 with a specific neutralizing antibody. This treatment caused a similar result in young mice to that observed in aged mice, with increased neutrophil recruitment to BAL and lungs, similar levels of proinflammatory cytokines, and an increase in some chemokines (CCL3 and CCL5). These data indicate that IL-10 plays an important functional role in regulating the recruitment of neutrophils into infected lungs. This heightened recruitment occurred despite a significant reduction in bacterial burden by 48 h postinfection, further suggesting a lack of resolution of inflammation in these aged animals. However, bacterial lung burden was not altered after anti-IL-10 treatment, despite the corresponding increase in neutrophils, comparable to the findings in aged mice following *S. pneumoniae* infection. This suggests that bacterial clearance may be independent of IL-10, whereas the extent of neutrophilic inflammation is dependent on IL-10. Previous reports have been somewhat conflicting regarding the role of IL-10 in host defense against *S. pneumoniae*. Some report that IL-10 actually impairs host defense against *S. pneumoniae* (50, 52), whereas others report that IL-10 is protective in models of septic peritonitis (49). Further studies have shown that the absence of IL-10 results in immunopathology independent of pathogen load (17, 21), an observation that the present study supports. These findings and the data reported herein indicate that the balance between host defense and the regulation of excessive inflammation is highly complex.

Chemokines play an instrumental role in directing leukocyte trafficking into sites of inflammation. CXCL1 (KC) is considered to be the archetypal neutrophil chemoattractant, which is the murine functional homologue of human CXCL8 (IL-8). Even though aged mice recruited higher numbers of neutrophils than young mice, there were no differences in the levels of CXCL1. This suggests that excessive neutrophil influx in aged mice may be dependent on other chemokines. Of the chemokines tested, CCL3, CCL4, CCL5, CCL11, CXCL9, and CXCL12 were all elevated in the lungs of aged mice, suggesting they might be involved in mediating the heightened inflammatory response of aged mice. Furthermore, inhibiting IL-10 resulted in an increase in CCL3 and CCL5, two of the chemokines that were increased in aged mice following pneumococcal infection. This would suggest that IL-10 modulates the expression of these chemokines following challenge with *S. pneumoniae* and that these chemokines may be important in mediating the enhanced inflammatory response of aged mice. IL-10 has previously been shown to modulate neutrophil and eosinophil recruitment in a mouse model of ovalbumin sensitization (58). In a mouse model of LPS-induced inflammation, IL-10 was shown to regulate the expression of CCL3 and CXCL2 and decrease neutrophil accumulation into air spaces (44). This likely reflects the dynamic relationship between various pro- and anti-inflammatory mediators during an innate immune response to a bacterial infection. The way in which the immune system responds to a microorganism is therefore a critical factor that determines the severity of disease (36).

Although the present study suggests that aged mice have a heightened innate immune response to bacterial infection, several questions regarding the role of IL-10 in this process, and the functional capacity of aged neutrophils, remain. For example, restoring the levels of IL-10 in aged animals to similar levels in young adult mice would provide important information regarding the role of this cytokine in regulating inflammation during the aging process. Extending these studies to strains of mice that are more susceptible to *S. pneumoniae* infection, such as C57BL/6 or CD1, may also help to delineate how IL-10 regulates the host response to this pathogen. Further studies on the microbicidal capacity of aged neutrophils would also be useful to determine whether these cells have reduced functionality in aged mice. In addition, studies designed to address the effect of IL-10 on neutrophil recruitment and on the regulation of the pulmonary chemokine network would be informative and should form the basis of future research activity in this area.

To summarize, we provide evidence that aged mice respond to *S. pneumoniae* infection with a heightened pulmonary innate immune response compared with young adult mice, represented by increased neutrophil recruitment, even though this did not seem to affect lung protective immunity. We further show that IL-10 production in response to *S. pneumoniae* is reduced in aged mice and is associated with increased chemokine production. These data support the notion that loss of IL-10 production with age, during an innate immune response within the lung, is associated with enhanced inflammatory responses to this common pathogen. This study also supports the immune dysregulation theory of aging, whereby normal regulatory components of the immune system, in this case IL-10, are lost, resulting in an underlying proinflammatory state (7). This study also supports the concept of inflammaging (14), whereby age-related diseases are often associated with chronic inflammation. A similar mechanism might underlie the greater degree of morbidity and mortality reported in elderly patients during *S. pneumoniae* pneumonia compared with younger subjects (6, 48), although we accept there are physiological differences between aged mice and elderly humans. As global demographics shift toward an aged population, understanding the mechanisms underlying the fine control of innate immune responses to *S. pneumoniae* during aging may reveal new molecular targets that could improve outcome in elderly individuals.

## GRANTS

This work was supported by funding from the Rosetrees Trust (Grant No. A420 to R. C. Chambers and A. E. Williams) and the NIHR UCLH Biomedical Research Centre (Grant No. BRC/76HI/RC to R. C. Chambers and A. E. Williams), The Wellcome Trust (Grant No. 097216/Z/11/Z to R. J. José and R. C. Chambers); and the NIHR UCLH Biomedical Research Centre (to J. S. Brown).

## DISCLOSURES

No conflicts of interest, financial or otherwise, are declared by the author(s).

## AUTHOR CONTRIBUTIONS

A.E.W. and R.C.C. conception and design of research; A.E.W. and R.J.J. performed experiments; A.E.W. analyzed data; A.E.W., R.J.J., J.S.B., and R.C.C. interpreted results of experiments; A.E.W. prepared figures; A.E.W., R.J.J., J.S.B., and R.C.C. drafted manuscript; A.E.W., J.S.B., and R.C.C. edited and revised manuscript; A.E.W., R.J.J., J.S.B., and R.C.C. approved final version of manuscript.

## References

[1] Agarwal S, Busse PJ Innate and adaptive immunosenescence. Ann Allergy Asthma Immunol 104: 183–190, 2010. 20377107 10.1016/j.anai.2009.11.009

[2] Bogaert D, De Groot R, Hermans PW Streptococcus pneumoniae colonisation: the key to pneumococcal disease. Lancet Infect Dis 4: 144–154, 2004. 14998500 10.1016/S1473-3099(04)00938-7

[3] Boyd AR, Shivshankar P, Jiang S, Berton MT, Orihuela CJ Age-related defects in TLR2 signaling diminish the cytokine response by alveolar macrophages during murine pneumococcal pneumonia. Exp Gerontol 47: 507–518, 2012. 22548913 10.1016/j.exger.2012.04.004 PMC3368096

[4] Brown JS Community-acquired pneumonia. Clin Med 12: 538–543, 2012. 23342408 10.7861/clinmedicine.12-6-538 PMC5922594

[5] Butcher SK, Chahal H, Nayak L, Sinclair A, Henriquez NV, Sapey E, O'Mahony D, Lord JM Senescence in innate immune responses: reduced neutrophil phagocytic capacity and CD16 expression in elderly humans. J Leukoc Biol 70: 881–886, 2001. 11739550

[6] Cabre M Pneumonia in the elderly. Curr Opin Pulm Med 15: 223–229, 2009. 19276811 10.1097/MCP.0b013e328326f571

[7] Castelo-Branco C, Soveral I The immune system and aging: a review. Gynecol Endocrinol 30: 16–22, 2014. 24219599 10.3109/09513590.2013.852531

[8] Castle SC Clinical relevance of age-related immune dysfunction. Clin Infect Dis 31: 578–585, 2000. 10987724 10.1086/313947

[9] Chelvarajan RL, Collins SM, Van Willigen JM, Bondada S The unresponsiveness of aged mice to polysaccharide antigens is a result of a defect in macrophage function. J Leukoc Biol 77: 503–512, 2005. 15629885 10.1189/jlb.0804449

[10] Chignard M, Balloy V Neutrophil recruitment and increased permeability during acute lung injury induced by lipopolysaccharide. Am J Physiol Lung Cell Mol Physiol 279: L1083–L1090, 2000. 11076798 10.1152/ajplung.2000.279.6.L1083

[11] Cilloniz C, Ewig S, Polverino E, Marcos MA, Esquinas C, Gabarrus A, Mensa J, Torres A Microbial aetiology of community-acquired pneumonia and its relation to severity. Thorax 66: 340–346, 2011. 21257985 10.1136/thx.2010.143982

[12] Douziech N, Seres I, Larbi A, Szikszay E, Roy PM, Arcand M, Dupuis G, Fulop T Jr. Modulation of human lymphocyte proliferative response with aging. Exp Gerontol 37: 369–387, 2002. 11772524 10.1016/s0531-5565(01)00204-2

[13] Emanuelli G, Lanzio M, Anfossi T, Romano S, Anfossi G, Calcamuggi G Influence of age on polymorphonuclear leukocytes in vitro: phagocytic activity in healthy human subjects. Gerontology 32: 308–316, 1986. 3582991 10.1159/000212809

[14] Franceschi C, Campisi J Chronic inflammation (inflammaging) and its potential contribution to age-associated diseases. J Gerontol A Biol Sci Med Sci 69, Suppl 1: S4–S9, 2014. 24833586 10.1093/gerona/glu057

[15] Fulop T Jr, Foris G, Worum I, Leovey A Age-dependent alterations of Fc gamma receptor-mediated effector functions of human polymorphonuclear leucocytes. Clin Exp Immunol 61: 425–432, 1985. 2994926 PMC1577299

[16] Gavazzi G, Krause KH Ageing and infection. Lancet Infect Dis 2: 659–666, 2002. 12409046 10.1016/s1473-3099(02)00437-1

[17] Gazzinelli RT, Wysocka M, Hieny S, Scharton-Kersten T, Cheever A, Kuhn R, Muller W, Trinchieri G, Sher A In the absence of endogenous IL-10, mice acutely infected with Toxoplasma gondii succumb to a lethal immune response dependent on CD4+ T cells and accompanied by overproduction of IL-12, IFN-gamma and TNF-alpha. J Immunol 157: 798–805, 1996. 8752931

[18] Gessner MA, Doran SF, Yu Z, Dunaway CW, Matalon S, Steele C Chlorine gas exposure increases susceptibility to invasive lung fungal infection. Am J Physiol Lung Cell Mol Physiol 304: L765–L773, 2013. 23564508 10.1152/ajplung.00030.2013 PMC3680763

[19] Ginaldi L, Loreto MF, Corsi MP, Modesti M, De Martinis M Immunosenescence and infectious diseases. Microbes Infect 3: 851–857, 2001. 11580980 10.1016/s1286-4579(01)01443-5

[20] Godbole G, Gant V Respiratory tract infections in the immunocompromised. Curr Opin Pulm Med 19: 244–250, 2013. 23508112 10.1097/MCP.0b013e32835f82a9

[21] Grunig G, Corry DB, Leach MW, Seymour BW, Kurup VP, Rennick DM Interleukin-10 is a natural suppressor of cytokine production and inflammation in a murine model of allergic bronchopulmonary aspergillosis. J Exp Med 185: 1089–1099, 1997. 9091582 10.1084/jem.185.6.1089 PMC2196229

[22] Gutierrez F, Masia M Improving outcomes of elderly patients with community-acquired pneumonia. Drugs Aging 25: 585–610, 2008. 18582147 10.2165/00002512-200825070-00005

[23] Guzzetta G, Kirschner D The roles of immune memory and aging in protective immunity and endogenous reactivation of tuberculosis. PLoS One 8: e60425, 2013. 23580062 10.1371/journal.pone.0060425 PMC3620273

[24] Hinojosa CA, Babu ASR, Rahman MM, Fernandes G, Boyd AR, Orihuela CJ Elevated A20 contributes to age-dependent macrophage dysfunction in the lungs. Exp Gerontol 54: 58–66, 2014. 24440463 10.1016/j.exger.2014.01.007 PMC3989429

[25] Hirano S Interaction of rat alveolar macrophages with pulmonary epithelial cells following exposure to lipopolysaccharide. Arch Toxicol 70: 230–236, 1996. 8825682 10.1007/s002040050265

[26] Kadioglu A, Andrew PW The innate immune response to pneumococcal lung infection: the untold story. Trends Immunol 25: 143–149, 2004. 15036042 10.1016/j.it.2003.12.006

[27] Kadioglu A, Weiser JN, Paton JC, Andrew PW The role of Streptococcus pneumoniae virulence factors in host respiratory colonization and disease. Nat Rev Microbiol 6: 288–301, 2008. 18340341 10.1038/nrmicro1871

[28] Kale S, Yende S, Kong L, Perkins A, Kellum JA, Newman AB, Vallejo AN, Angus DC The effects of age on inflammatory and coagulation-fibrinolysis response in patients hospitalized for pneumonia. PLoS One 5: e13852, 2010. 21085465 10.1371/journal.pone.0013852 PMC2973976

[29] Kaplan V, Angus DC, Griffin MF, Clermont G, Scott WR, Linde-Zwirble WT Hospitalized community-acquired pneumonia in the elderly: age- and sex-related patterns of care and outcome in the United States. Am J Respir Crit Care Med 165: 766–772, 2002. 11897642 10.1164/ajrccm.165.6.2103038

[30] Kolaczkowska E, Kubes P Neutrophil recruitment and function in health and inflammation. Nat Rev Immunol 13: 159–175, 2013. 23435331 10.1038/nri3399

[31] Larbi A, Douziech N, Dupuis G, Khalil A, Pelletier H, Guerard KP, Fulop T Jr. Age-associated alterations in the recruitment of signal-transduction proteins to lipid rafts in human T lymphocytes. J Leukoc Biol 75: 373–381, 2004. 14657209 10.1189/jlb.0703319

[32] Lynch JP III, Zhanel GG Streptococcus pneumoniae: epidemiology, risk factors, and strategies for prevention. Semin Respir Crit Care Med 30: 189–209, 2009. 19296419 10.1055/s-0029-1202938

[33] Lynch JP III, Zhanel GG Streptococcus pneumoniae: epidemiology and risk factors, evolution of antimicrobial resistance, and impact of vaccines. Curr Opin Pulm Med 16: 217–225, 2010. 20375783 10.1097/MCP.0b013e3283385653

[34] Milbrandt EB, Reade MC, Lee M, Shook SL, Angus DC, Kong L, Carter M, Yealy DM, Kellum JA Prevalence and significance of coagulation abnormalities in community-acquired pneumonia. Mol Med 15: 438–445, 2009. 19753144 10.2119/molmed.2009.00091 PMC2743205

[35] Naylor K, Li G, Vallejo AN, Lee WW, Koetz K, Bryl E, Witkowski J, Fulbright J, Weyand CM, Goronzy JJ The influence of age on T cell generation and TCR diversity. J Immunol 174: 7446–7452, 2005. 15905594 10.4049/jimmunol.174.11.7446

[36] Pirofski LA, Casadevall A Q and A: What is a pathogen? A question that begs the point. BMC Biol 10: 6, 2012. 22293325 10.1186/1741-7007-10-6 PMC3269390

[37] Pitsiou GG, Kioumis IP Pneumococcal vaccination in adults: does it really work? Respir Med 105: 1776–1783, 2011. 21816596 10.1016/j.rmed.2011.07.008

[38] Rajagopalan S, Yoshikawa TT Tuberculosis in the elderly. Z Gerontol Geriatr 33: 374–380, 2000. 11130191 10.1007/s003910070034

[39] Romero-Steiner S, Musher DM, Cetron MS, Pais LB, Groover JE, Fiore AE, Plikaytis BD, Carlone GM Reduction in functional antibody activity against Streptococcus pneumoniae in vaccinated elderly individuals highly correlates with decreased IgG antibody avidity. Clin Infect Dis 29: 281–288, 1999. 10476727 10.1086/520200

[40] Sanders KM, Marras TK, Chan CK Pneumonia severity index in the immunocompromised. Can Respir J 13: 89–93, 2006. 16550266 10.1155/2006/195464 PMC2539011

[41] Santiago AF, Alves AC, Oliveira RP, Fernandes RM, Paula-Silva J, Assis FA, Carvalho CR, Weiner HL, Faria AM Aging correlates with reduction in regulatory-type cytokines and T cells in the gut mucosa. Immunobiology 216: 1085–1093, 2011. 21676485 10.1016/j.imbio.2011.05.007 PMC3206609

[42] Saraiva M, O'Garra A The regulation of IL-10 production by immune cells. Nat Rev Immunol 10: 170–181, 2010. 20154735 10.1038/nri2711

[43] Schmitt V, Rink L, Uciechowski P The Th17/Treg balance is disturbed during aging. Exp Gerontol 48: 1379–1386, 2013. 24055797 10.1016/j.exger.2013.09.003

[44] Shanley TP, Vasi N, Denenberg A Regulation of chemokine expression by IL-10 in lung inflammation. Cytokine 12: 1054–1064, 2000. 10880252 10.1006/cyto.1999.0655

[45] Siegrist CA, Aspinall R B-cell responses to vaccination at the extremes of age. Nat Rev Immunol 9: 185–194, 2009. 19240757 10.1038/nri2508

[46] Simell B, Vuorela A, Ekstrom N, Palmu A, Reunanen A, Meri S, Kayhty H, Vakevainen M Aging reduces the functionality of anti-pneumococcal antibodies and the killing of Streptococcus pneumoniae by neutrophil phagocytosis. Vaccine 29: 1929–1934, 2011. 21236231 10.1016/j.vaccine.2010.12.121

[47] Simon RH, DeHart PD, Todd RF III Neutrophil-induced injury of rat pulmonary alveolar epithelial cells. J Clin Invest 78: 1375–1386, 1986. 3771800 10.1172/JCI112724 PMC423838

[48] van den Biggelaar AH, Huizinga TW, de Craen AJ, Gussekloo J, Heijmans BT, Frolich M, Westendorp RG Impaired innate immunity predicts frailty in old age. The Leiden 85-plus study. Exp Gerontol 39: 1407–1414, 2004. 15489064 10.1016/j.exger.2004.06.009

[49] van der Poll T, Marchant A, Buurman WA, Berman L, Keogh CV, Lazarus DD, Nguyen L, Goldman M, Moldawer LL, Lowry SF Endogenous IL-10 protects mice from death during septic peritonitis. J Immunol 155: 5397–5401, 1995. 7594556

[50] van der Poll T, Marchant A, Keogh CV, Goldman M, Lowry SF Interleukin-10 impairs host defense in murine pneumococcal pneumonia. J Infect Dis 174: 994–1000, 1996. 8896500 10.1093/infdis/174.5.994

[51] van der Poll T, Opal SM Pathogenesis, treatment, and prevention of pneumococcal pneumonia. Lancet 374: 1543–1556, 2009. 19880020 10.1016/S0140-6736(09)61114-4

[52] van der Sluijs KF, van Elden LJ, Nijhuis M, Schuurman R, Pater JM, Florquin S, Goldman M, Jansen HM, Lutter R, van der Poll T IL-10 is an important mediator of the enhanced susceptibility to pneumococcal pneumonia after influenza infection. J Immunol 172: 7603–7609, 2004. 15187140 10.4049/jimmunol.172.12.7603

[53] Wenisch C, Patruta S, Daxbock F, Krause R, Horl W Effect of age on human neutrophil function. J Leukoc Biol 67: 40–45, 2000. 10647996 10.1002/jlb.67.1.40

[54] Williams AE, Chambers RC The mercurial nature of neutrophils: still an enigma in ARDS? Am J Physiol Lung Cell Mol Physiol 306: L217–L230, 2014. 24318116 10.1152/ajplung.00311.2013 PMC3920201

[55] Xu X, Jackson PL, Tanner S, Hardison MT, Abdul RM, Blalock JE, Gaggar A A self-propagating matrix metalloprotease-9 (MMP-9) dependent cycle of chronic neutrophilic inflammation. PLoS One 6: e15781, 2011. 21249198 10.1371/journal.pone.0015781 PMC3020950

[56] Zemans RL, Briones N, Campbell M, McClendon J, Young SK, Suzuki T, Yang IV, De Langhe S, Reynolds SD, Mason RJ, Kahn M, Henson PM, Colgan SP, Downey GP Neutrophil transmigration triggers repair of the lung epithelium via beta-catenin signaling. Proc Natl Acad Sci USA 108: 15990–15995, 2011. 21880956 10.1073/pnas.1110144108 PMC3179042

[57] Zemans RL, Colgan SP, Downey GP Transepithelial migration of neutrophils: mechanisms and implications for acute lung injury. Am J Respir Cell Mol Biol 40: 519–535, 2009. 18978300 10.1165/rcmb.2008-0348TR PMC2677434

[58] Zuany-Amorim C, Haile S, Leduc D, Dumarey C, Huerre M, Vargaftig BB, Pretolani M Interleukin-10 inhibits antigen-induced cellular recruitment into the airways of sensitized mice. J Clin Invest 95: 2644–2651, 1995. 7769104 10.1172/JCI117966 PMC295947

